# Structural changes and molecular mechanism study on the inhibitory activity of epigallocatechin against α-glucosidase and α-amylase

**DOI:** 10.3389/fnut.2022.948027

**Published:** 2022-11-09

**Authors:** Ziyi Man, Yi Feng, Jibo Xiao, Hailong Yang, Xiangting Wu

**Affiliations:** College of Life and Environmental Sciences, Wenzhou University, Wenzhou, China

**Keywords:** epigallocatechin, α-glucosidase, α-amylase, non-competitive inhibition, intermolecular hydrogen bonds, hydrophobic interaction

## Abstract

In this study, the inhibition and mechanism of epigallocatechin (EGC) on two key glycoside hydrolases (α-glucosidase, α-amylase) were explored from the molecular structure level. The chemical structure of EGC was characterized by X-ray diffraction, Fourier transform infrared (FTIR) spectroscopy, and proton nuclear magnetic resonance spectroscopy. EGC’s inhibition on these enzymes was colorimetrically determined. The effects of EGC on the chemical structure and spatial configuration of the enzymes were explored *via* FTIR spectroscopy, fluorescence spectroscopy, and molecular docking techniques. The results showed that EGC exhibited the inhibition of α-glucosidase and α-amylase in a non-competitive manner, showing a continuous upward trend as EGC’s concentration increased. There was a fluorescence quenching effect of EGC on α-glucosidase and α-amylase. Molecular docking confirmed that EGC can bind to amino acid residues in the enzyme through intermolecular hydrogen bonds and hydrophobic interactions, resulting in the changed chemical structure and spatial conformation of the enzymes. This decreased enzyme activity. This result suggested that EGC has the potential to inhibit two key glycoside hydrolases, and it would be beneficial to incorporate EGC into functional foods for diabetics.

## Introduction

Diabetes mellitus (DM), a chronic illness worldwide, is characterized by chronic hyperglycemia and dyslipidemia (1). A sharp increase in the number of people with DM worldwide exists due to lifestyle changes and eating habits. Ingestion of high-carbohydrate or high-sugar foods can cause elevated postprandial blood glucose (PBG) that leads to II-DM (2). The two most important glycoside hydrolases affecting PBG levels, α-glucosidase and α-amylase, play a key role in carbohydrate absorption and digestion (3). PBG can be maintained at a normal level by inhibiting α-glucosidase and α-amylase activities, delaying the absorption of carbohydrates, or controlling the contact process between carbohydrates and enzymes (4). Currently, clinical drugs used for treating DM *via* glycoside hydrolases inhibition are mainly Acarbose and Voglibose (5). Although these drugs can effectively control PBG increases, their long-term use causes a series of side effects, such as nausea, vomiting, flatulence, diarrhea, and gradually increased drug resistance (6). Increasing evidence shows that many tea extracts inhibit α-glucosidase and α-amylase, thereby showing hypoglycemic activity (7–9). Therefore, finding safe and effective α-glucosidase or α-amylase inhibitors from natural plant resources is a key task in the fields of food, biology, and medicine.

Epigallocatechin (EGC), natural hydrolyzed catechin monomers from tea, is one of the most abundant, bioactive, and non-toxic polyphenols in tea (10). Modern pharmacological research has shown that plant polyphenols have antioxidants and regulate blood sugar and lipid levels because of their several -OH and cyclic structure (11). Wu (12) reported that epicatechin gallate’s (ECG) inhibition mechanism for α-glucosidase and α-amylase is related to Gln63 and Asp197 of α-amylase and Lys156, Ser157, Arg315, and Asp352 of α-glucosidase. Zhao (13) reported that the combination of hawthorn polyphenols, D-chiro-inositol, and ECG could improve insulin resistance and reduce fasting blood glucose and hepatic gluconeogenesis by downregulating PI3K/Akt/FOXO1-mediated PEPCK and G6-Pase and upregulating PI3K/Akt/GSK3-mediated hepatic glycogen synthase GS activation in the liver. Currently, extensive studies have been reported on tea extracts as glycoside hydrolases inhibitors, such as optimization of the extract preparation process, selection of extraction solvents, and comparison of inhibitory activity of different tea types and sources (14–16). Although catechin-containing tea extracts have been used as glycoside hydrolases inhibitors, few reports exist on purified catechin monomers as inhibitors. Additionally, few reports exist on the inhibition of α-glucosidase and α-amylase *via* catechin monomer EGC *in vitro*, and the mechanism of action affecting it is not clear. Therefore, it is a necessary aid in systematically exploring the inhibitory mechanism of polyphenols against glycoside hydrolases by studying the molecular structure changes and interaction mechanism between polyphenols and enzymes.

In this work, EGC’s chemical structure and types of inhibitory effects on α-glucosidase and α-amylase were analyzed. EGC changes in the chemical and spatial structures of α-glucosidase and α-amylase were observed *via* Fourier transform infrared (FTIR) spectroscopy and fluorescence spectroscopy. The molecular mechanism of EGC’s inhibition interacting with α-glucosidase and α-amylase was preliminarily elucidated. Completion of this study gives a better understanding of the possible effect and mechanism of tea polyphenol and catechin for II-DM’s possible prevention.

## Materials and methods

### Chemicals and materials

Epigallocatechin (≥ 95% purity) was obtained from Shennong Bio-Technology Co., Ltd. (Shaanxi, China). α-glucosidase (50 U/mg, source: *Saccharomyces cerevisiae*), α-amylase (35 U/mg, source: Porcine pancreas), 4-Nitrophenyl α-D-galacto-pyran-oside (PNPG) (≥ 99% purity), and acarbose (≥ 98% purity) were purchased from Macklyn Bio-Technology Co., Ltd. (Shanghai, China). KB_*r*_ (spectral grade) and starch soluble were purchased from Aladdin Industrial Co., Ltd. (Shanghai, China). DNS (Ghose method) was purchased from Beijing Solarbio Science & Technology Co., Ltd (Beijing, China). All other chemical reagents used were of analytical grade.

### Epigallocatechin’s structural characterization

#### X-ray diffraction measurements

The crystallinity of EGC was determined *via* an X-ray diffractometer (Rigaku SmartLab SE, JNP). Cu–Kα radiation from a sealed tube was used. An appropriate sample amount was applied to the groove on the slide, and data were collected in the 2θ range of 5°–90° with a step of 10°/min (17).

#### Fourier transform infrared measurements

One mg of the sample was mixed with 300 mg KB_*r*_, compressed into a tablet, and tested using a Nicolet iN 10 MX FTIR spectrophotometer (Thermo Scientific, USA). FTIR data was obtained by collecting data in the range of 400–4,000 cm^–1^. KB_*r*_ was the background during the measurement. The spectral resolution was set to 4 cm^–1^ at a rate of 16 nm/s (18).

#### Proton nuclear magnetic resonance (^1^H NMR) measurements

Fifteen mg of the EGC sample was transferred to a 2 ml Eppendorf tube. Next, 0.5 ml of NMR grade dimethyl sulfoxide (DMSO) solvent was added to dissolve the compound *via* sonication. Finally, the clarified solution was transferred to 5 mm NMR tubes, and data were obtained using an AVANCE3 AV500 NMR spectrometer (Bruker, Germany). Tetramethylsilane (TMS) was the relative internal standard, and all chemical shifts were in ppm (19).

### Assay of inhibition activity on α-glucosidase and α-amylase

The assay of inhibition activity on α-glucosidase and α-amylase was performed according to the method of literature (20, 21) with slight modification.

The α-glucosidase solution (40 μL, 0.2 mol/L) and different concentrations of EGC solution (40 μL, 0.6, 0.8, 1.0, 1.2, 1.4 mg/ml, and 1.6 mg/ml) were cultured at 37°C for 10 min to determine α-glucosidase inhibition activity, and PNPG (20 μL, 0.02 mol/L) was added. Solutions containing PNPG were incubated at 37°C for 30 min. Following this, Na_2_CO_3_ (50 μL, 1 mol/L) was added to terminate the reaction. The reaction solution’s absorbance was measured at 405 nm *via* the Synergy H4 Hybrid microplate reader (BioTek, America). Triplicate samples for each test were created. The samples’ inhibitory activity was described by the inhibition rate and half-maximal inhibitory concentration (IC_50_) values. Table 1 shows the specific measurements. The inhibition rate was calculated according to the formula of equation 1.

**TABLE 1:** Inhibition experiment of epigallocatechin on α-glucosidase and α-amylase.

	Samples								

	α-glucosidase inhibition assay				α-amylase inhibition assay				
		
	0.2 mol/L α-glucosidase	EGC solution	0.2 mol/L PBS	0.02 mol/L PNPG	0.5 mg/ml α-amylase	EGC solution	starch solution (1%)	DNS	0.2 mol/L PBS
A_0_	40 μL	40 μL	–	20 μL	100 μL	100 μL	250 μL	200 μL	–
A_1_	–	40 μL	40 μL	20 μL	–	100 μL	250 μL	200 μL	100 μL
A_2_	40 μL	–	40 μL	20 μL	100 μL	–	250 μL	200 μL	100 μL
A_3_	–	–	40 μL	20 μL	–	–	250 μL	200 μL	100 μL

The α-amylase solutions (100 μL, 5 mg/ml) and EGC solutions (100 μL, 0.6, 0.8, 1.0, 1.2, 1.4, and 1.6 mg/ml) of different concentrations were incubated at 37°C for 10 min to determine α-amylase inhibition activity. The 1% starch solution (250 μL) was added and incubated at 37°C for 10 min. The DNS reagent (200 μL) was added to the reaction solution after incubation. The mixed solution was incubated in water at 100°C for 15 min and cooled to 20°C. Finally, 200 μL distilled water diluted the reaction solution. The absorbance of the reaction solution was measured at 540 nm *via* the Synergy H4 Hybrid microplate reader (BioTek, America). Triplicate samples for each test were created. The samples’ inhibitory activity was described *via* the inhibition rate and IC_50_ values. Table 1 shows the specific measurements. The inhibition rate was calculated according to Eq. 1:


(1) $$Inhibitionrate\left(\%\right)=\left(1-\frac{A_{0}-A_{1}}{A_{2}-A_{3}}\right)\times 100\%$$

### Determination of inhibition kinetics of epigallocatechin against α-glucosidase and α-amylase

Epigallocatechin’s inhibition kinetics against α-glucosidase and α-amylase were investigated according to the method of literature (22). The assay was carried out in the same way as in section “Assay of inhibition activity on α-glucosidase and α-amylase.” The Lineweaver–Burk plot of EGC at different concentrations was drawn by taking the 1/[*S*] as the *X*-axis and the 1/*v* as the *Y*-axis. The inhibition mode was determined *via* the kinetic Cornish-Bowden (Eq. 2).

Cornish-Bowden equation:


(2) $$\frac{1}{V}=\frac{K_{m}}{V_{\max}}\,\left(1+\frac{\left[C\right]}{K_{i\,c}}\right)\,\frac{1}{\left[S\right]}+\frac{1}{V_{\max}}\,\left(1+\frac{\left[C\right]}{K_{i\,u}}\right)$$

where *V* (mol/L/min) is the enzymatic reaction rate. *V*_*max*_ (mol/L/min) is the maximum reaction rate. *K*_*m*_ (mg/ml) is the Michaelis constant. [*C*] (mg/ml) is the concentration of EGC. *K*_*ic*_ is the competitive inhibition constant. *K*_*iu*_ is the non-competitive inhibition constant. [*S*] (mg/ml) is the concentrations of starch or PNPG solution.

### Fluorescence spectroscopy measurements

The F-7000 fluorescence photometer (Hitachi, Japan) tested the fluorescence spectrum of the sample. Different concentrations of EGC solution, α-glucosidase solution (0.2 mol/L), and α-amylase solution (0.5 mg/ml) were prepared in phosphate*-*buffered saline (PBS) (0.2 mol/L, pH 6.8). The EGC solution was thoroughly mixed with the α-glucosidase and α-amylase solutions (V/V = 1/1) and was incubated at 37°C for 10 min before its fluorescence absorbance was measured. The excitation wavelength was 280 nm, and the scanning range was 290–500 nm, with a scanning interval of 5 nm (23).

### Molecular docking simulation of α-glucosidase, α-amylase, and epigallocatechin

Molecular docking simulation was performed *via* the Autodock Vina software (24). The 3-dimensional (3D) structures of α-glucosidase (PDB: 3A4A) and α-amylase (PDB: 1HNY) obtained from the Protein Data Bank^1^ were elected as receptors in the docking process (25). The 3D structures of EGC available in PubChem^2^ were considered ligands in the docking process (26). Before molecular docking began, protein molecules and water molecules in the enzymes’ crystal structure were removed (26). Molecular docking results were imaged with the ChimeraX and LigPlus software, and the docking sites, hydrogen bonds, and hydrophobic interaction were visualized.

### Statistical analysis

The data were presented as average-value ± SD. SPSS.26.0 was used for the significant analysis of the results. *P* < 0.05 denoted a significant difference.

## Results and discussion

### Structural characterization of epigallocatechin

**FIGURE 1:**
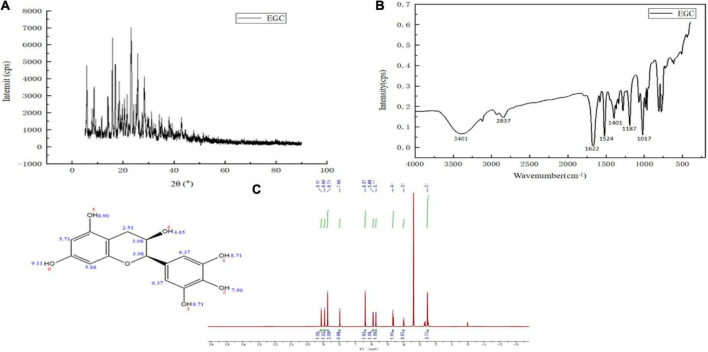
Chemical structural characterization of epigallocatechin: X-ray diffraction (XRD) **(A)**, Fourier transform infrared (FTIR) **(B)**, and Proton nuclear magnetic resonance (^1^H NMR) **(C)**.

The structure characterization of EGC was obtained by various strategies, including X-ray diffraction (XRD), FTIR, and ^1^H NMR. Figure 1A shows the XRD of EGC. Crystals generally exhibit a sharp and narrow characteristic peak, while amorphous crystals exhibit a broad and diffuse diffraction halo (27). EGC’s XRD results showed many sharp diffraction peaks in the range of 5°–40°, demonstrating EGC’s crystalline nature. Figure 1B shows the result of EGC’s FTIR. Phenolic compounds consisted of one or more benzene rings, and at least one hydroxyl group directly attached to them (28). EGC exhibits common bands associated with these structures. The region between 4,000 and 2,500 cm^–1^ mainly contained broad bands associated with the -OH stretching vibration and the aromatic C–H stretching vibration (28). The range from 1,800 to 700 cm^–1^ was called the “fingerprint region” and contained rich structural information (28). Additionally, the phenolic compounds’ structures are complex, and their environment affected the vibration-related peaks (29). The stretching vibration range of C=C was generally 1,625–1,430 cm^–1^ (29). Aromatic six-membered rings usually exhibited two or three bands, with the strongest usually around 1,500 cm^–1^ (30). The -OH deformation and C-O stretching vibrations’ interaction was observed around 1,390–1,330 cm^–1^ and 1,260–1,180 cm^–1^ (31). Table 2 details the EGC’s FTIR spectroscopy information. Figure 1C shows EGC’s ^1^H NMR. EGC’s signals in ^1^H NMR contained more active hydrogen, so its spin system was hard to observe compared with other plant extracts (32). The signals at 7.96, 8.90, 9.11, and 4.65 ppm belong to -OH on the EGC molecule, which is more likely to form O-H⋅⋅⋅O hydrogen bonds (32). The EGC molecule was a cyclic structure with active groups, such as hydroxyl and phenolic hydroxyl groups, similar to other polyphenolic compounds. Therefore, we speculate that these active sites in EGC could more likely be combined with amino acid residues in the enzyme through hydrogen bonds, thereby changing the enzyme structure or forming more stable complexes.

**TABLE 2:** Functional groups of chemical compounds in epigallocatechin.

Functional group	Wavenumber (cm^–1^)
-OH	3403
C=C	1625–1430
Aromatic six-membered rings	1541–1558
	1517–1539
	1501–1521
C-H-O	1473–1502
-OH deformation	1323–1406
	1298–1274
C-O	1260–1246
	1146–1207
C-C	1099–1119
C-H	1015–1064
	982–1003
C-H deformation	870–896
	789–825

### Analysis of the inhibitory effect on α-glucosidase and α-amylase *via* epigallocatechin

**FIGURE 2:**
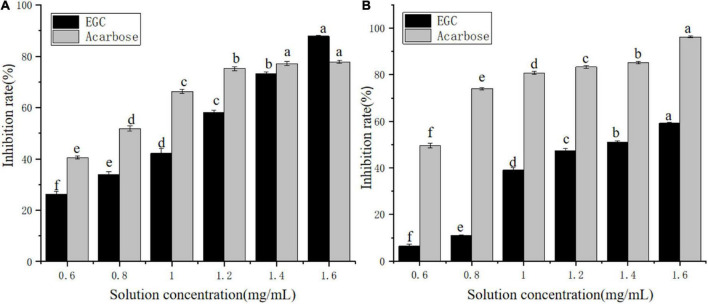
Inhibition effects of epigallocatechin on α-glucosidase **(A)** and α-amylase **(B)** (error bars represent standard deviation and different letters indicate significant differences at *p* < 0.05).

Currently, phenolic/flavonoid/catechin compounds extracted from tea extracts have been considered natural carbohydrate digestive enzyme inhibitors that can be applied in II-DM’s prevention or treatment (33–35). Inhibiting α-glucosidase and α-amylase’s activity is an effective method to control II-DM (36). Figure 2 shows the inhibitory effect of the EGC solution with different concentrations on α-glucosidase (Figure 2A) and α-amylase (Figure 2B) with a significant upward trend, and the inhibition rates reached 87.87 and 59.20%, respectively, within the tested concentration range. This indicated that EGC inhibited α-glucosidase and α-amylase’s activities in a concentration-dependent manner. Similar concentration-dependent inhibition effects were observed for the acarbose. When the inhibitors’ concentration was 1.6 mg/ml, EGC showed stronger α-glucosidase inhibitory activity compared to acarbose. However, EGC’s inhibitory effect on these enzymes was still lower compared to acarbose. Additionally, the EGC’s IC_50_ values calculated through the multivariant nonlinear regression were 1.04 ± 0.006 mg/ml and 1.36 ± 0.006 mg/ml, respectively, which were higher than the IC_50_ values of acarbose (0.71 ± 0.27 mg/ml and 0.37 ± 0.31 mg/ml) under the same conditions. This also indicated that the acarbose presented a greater potent inhibitory effect on α-glucosidase and α-amylase compared to EGC. Conversely, compared with the inhibitory effect of other tea extracts and catechins monomers on α-glucosidase and α-amylase activities. For example, the IC_50_ value of the ethanolic extract of partridge tea on α-amylase was 2.56 ± 0.35 mg/m (37). The IC_50_ value of α-glucosidase inhibition of green tea extract at 100°C was 6.31 ± 0.26 mg/ml (38). ECG’s IC_50_ values for the inhibition of α-amylase and α-glucosidase in mixed-type manners were 45.30 ± 0.22 and 4.03 ± 0.01 μg/ml, respectively (12). The catechins’ IC_50_ value for the inhibition of C-terminal maltase-glucoamylase was 7.8 ± 4.0 μg/ml (39). Thus, compared with mixed tea extracts, EGC showed stronger α-amylase and α-glucosidase inhibitory activities. However, compared with catechin monomers, EGC did not show the strongest α-amylase and α-glucosidase inhibitory activities. It may be because the Ghose method was only a rough method for determining the reduction of sugars and total sugars, and the measurement results are greatly affected by the reaction system and sample characterizations (40). Additionally, EGC has the advantages of being safe and having smaller side effects. It is more suitable for long-term blood sugar drugs (5, 8) and long-term control of blood glucose. These results suggest that EGC is a potent alternative α-glucosidase and α-amylase inhibitor with potential value in the production of hypoglycemic drugs and functional foods.

### Kinetic analysis of the inhibition of α-glucosidase and α-amylase *via* epigallocatechin

**FIGURE 3:**
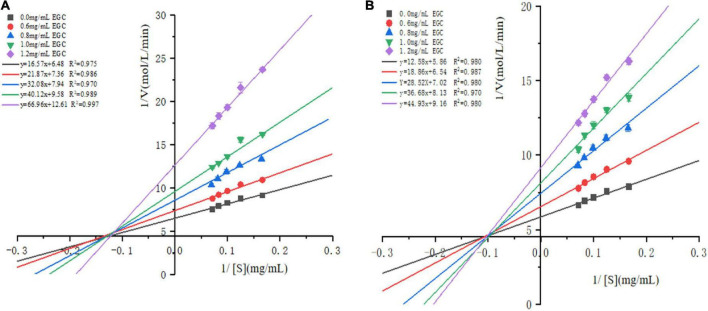
Lineweaver–Burk plots for inhibition of α-glucosidase and α-amylase by epigallocatechin with different concentrations.

The Lineweaver–Burk plots based on the kinetic Cornish-Bowden equation (Eq. 2) were established to define the inhibition mode. Figure 3 shows that in increasing the concentration of EGC, both the slopes and intercepts (1/*V*_*max*_) at the *Y*-axis of the enzymatic reaction rate line of EGC to α-glucosidase (Figure 3A) and α-amylase (Figure 3B) increased, which is the maximum reaction rate (*V*_*max*_) when decreased. At all EGC concentrations tested, the intercepts of the line on the *X*-axis (-1/*K*_*m*_) remained unchanged. The Michaelis constant (*K*_*m*_) remained constant regardless of EGC’s concentration. This suggests that EGC’s inhibition on the enzyme is independent of the substrate concentration, so EGC was a non-competitive inhibitor on α-glucosidase and α-amylase (41). Therefore, the inhibitory mechanism may be as follows: EGC combined with amino acid residues outside the active center of the enzyme changes the structure of the enzyme, subsequently decreasing the related carbohydrates decomposition rate and maintaining the stable PBG level.

### Epigallocatechin’s effect on the chemical structures of α-glucosidase and α-amylase

**FIGURE 4:**
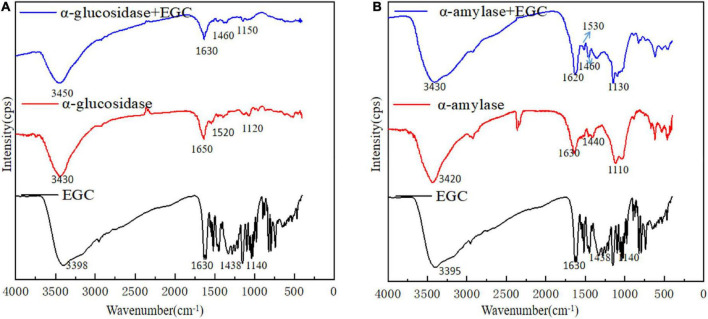
Fourier transform infrared (FTIR) spectra of different samples [**A**: epigallocatechin (EGC), α-glucosidase, α-glucosidase + EGC, **B**: EGC, α-amylase, α-amylase + EGC].

Fourier transform infrared plays an important role in analyzing chemical structural changes. Figure 4 shows EGC’s effects on the chemical structures of α-glucosidase (Figure 4A) and α-amylase (Figure 4B). Adding EGC had little effect on the FTIR spectra of α-glucosidase and α-amylase, suggesting no new covalent bonds in this structure. A similar phenomenon was observed in Jiang’s (42) research on chlorogenic acid’s effect on whey protein and casein. The absorption peak near 3,401 cm^–1^ was mainly due to the -OH stretching vibration. However, after EGC’s introduction, the characteristic peaks of -OH in the two EGC-enzyme complexes showed a slight blue shift, indicating the existence of hydrogen bonds in the structure. The amide I (1,600 cm–1,700 cm^–*l*^) originated from the C=O stretching vibration, amide II (1,400 cm–1,600 cm^–*l*^) originated from N-H, C-N stretching vibration, and amide III (1,100 cm–1,300 cm^–*l*^) originated from the C-O-C stretching vibration (43). After introducing EGC in the FTIR spectrum, the positions and peak shapes of amide I, amide II, and amide III changed, suggesting that EGC’s binding sites and enzymes may be C=O, C-O-C, C-N, and N-H. Amide III correlated with the protein structure, and the change meant the change of the protein structure (44). Therefore, EGC may be combined with α-glucosidase and α-amylase through intermolecular hydrogen bonds, changing the chemical structure and spatial conformation of the enzyme, leading varied to enzyme activity. This structural change produced by hydrogen bonds was still present in EGC’s catabolism, showing that EGC as an α-glucosidase and α-amylase inhibitor in food or medicine will not change its inhibitory effect due to molecular structure changes.

### Epigallocatechin’s fluorescence quenching effect on α-glucosidase and α-amylase

**FIGURE 5:**
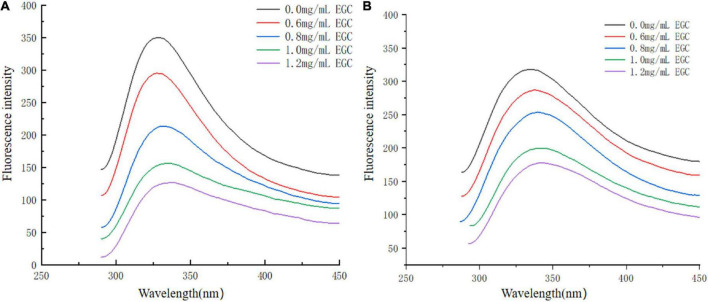
Fluorescence spectra of α-glucosidase **(A)** and α-amylase **(B)** treated with different concentrations of epigallocatechin.

Fluorescence spectroscopy experiments were used to investigate the interaction between EGC and α-glucosidase and α-amylase. At a certain excitation wavelength, the presence of tryptophan (Trp), tyrosine (Tyr), and phenylalanine (Phe) can cause fluorescent properties in α-glucosidase and α-amylase (45). Trp and Tyr residues are excited at 280 nm, and the microenvironment in which these residues are located affects fluorescence intensity and position (46). Figure 5 shows the fluorescence spectra of α-glucosidase (Figure 5A) and α-amylase (Figure 5B) with different EGC concentrations. EGC has a fluorescence quenching effect on α-glucosidase and α-amylase, and the fluorescence spectra show intrinsic peaks around 340 nm. With EGC’s increased concentration, the fluorescence intensity of α-glucosidase and α-amylase gradually decreased, and the maximum emission wavelength red-shift slightly, making α-amylase’s change more obvious. This concentration-dependent relationship showed EGC’s interaction with α-glucosidase and α-amylase (47). The red shift in the maximum emission wavelength in the fluorescence spectrum meant a change in part of the protein’s structure, which may result from EGC’s interaction with α-glucosidase and α-amylase (22). As the microenvironment changed from hydrophilic to hydrophobic, the protein structure unfolded and finally produced a fluorescence quenching effect. Therefore, we speculate that EGC may bind to Trp and Tyr residues in the enzyme through hydrogen bonding or hydrophobic interactions, causing changes in enzyme conformation and polarity and inhibiting enzyme activity.

### Molecular docking analysis

**FIGURE 6:**
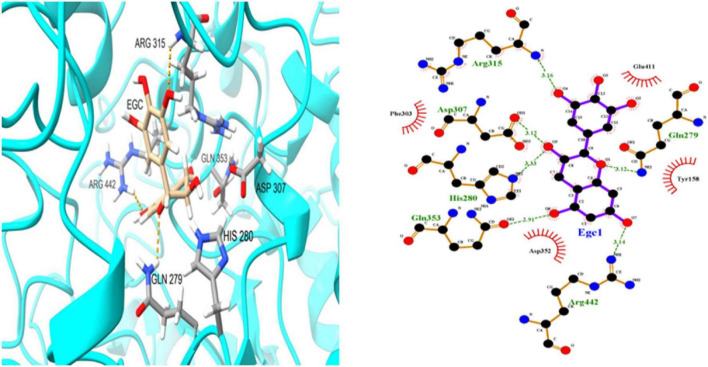
Molecular docking simulation diagrams of α-glucosidase and epigallocatechin (in 2-dimensional graphic, the green dotted line represents the hydrogen bond, and the red arc represents a hydrophobic interaction).

**FIGURE 7:**
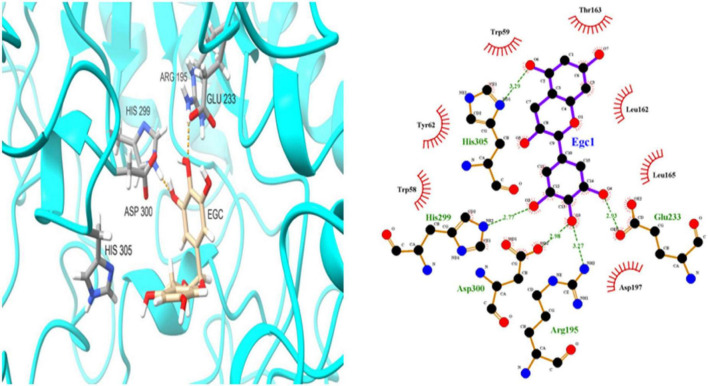
Molecular docking simulation diagrams of α-amylase and epigallocatechin (in the 2-dimensional graphic, the green dotted line represents the hydrogen bond, and the red arc represents a hydrophobic interaction).

To further investigate EGC’s interaction with α-glucosidase and α-amylase at the molecular level, molecular docking technology was used. Figures 6, 7 show the conformations when α-glucosidase (Figure 6) and α-amylase (Figure 7) interacted with EGC under the lowest energy, calculated by Autodock Vina. The Autodock Vina analysis showed that EGC is primarily bound to α-glucosidase and α-amylase through hydrogen bonding and hydrophobic interactions. EGC had lower binding energy with α-glucosidase (−8.383 kCal/mol) than with α-amylase (−7.235 kCal/mol). This showed that EGC inhibition of α-glucosidase was better than its inhibition of α-amylase, and the result was consistent with part 3.2. Figure 6 shows that the main sites of EGC binding to α-glucosidase were Arg135, Asp307, Gln279, His280, Gln353, Arg442, Glu411, Phe303, Try158, and Asp352. The complete hydrogen bonds in these sites were formed among Gln279, Arg315, and Arg442. Meanwhile, EGC and Glu411, Phe303, Try158, and Asp352 are mainly through hydrophobic interactions. Figure 7 shows that the main sites of EGC binding to α-amylase were His305, His229, Glu233, Asp300, Arg195, Thr163, Trp95, Tyr62, Leu162, Trp58, Leu165, and Asp197. In these sites, complete hydrogen bonds were formed among Arg195 and His299, along with other amino acids bound to EGC through hydrophobic interactions. In the molecular docking site of the two enzymes with EGC, the hydrogen bonds formed by His280, Asp307, and Gln353 on α-glucosidase and Glu233, Asp300, and His305 on α-amylase only meet the distance requirements. Therefore, the hydrogen bonds formed were incomplete. In reality, binding was heterogeneous and dynamic, so these incomplete hydrogen bonds were possible. Hydrogen bonding and hydrophobic interactions generally played an important role in maintaining the protein structure (25, 26). This also suggests that EGC’s involvement affected the enzyme’s spatial structure, ultimately leading to a change in enzyme activity, which agreed with fluorescence test’s consequences.

## Conclusion

In this study, spectroscopic techniques and molecular docking were used to investigate EGC’s interaction with two key glycoside hydrolases. The results showed that EGC had a non-competitive inhibitory effect on α-glucosidase and α-amylase, and the inhibition was mainly achieved by changing the enzyme’s chemical structure and spatial conformation through hydrogen bonding and hydrophobic interactions. The -OH in EGC could be combined with elements N, H, and O in the two enzymes through hydrogen bonds and combined with the non-polar groups in the enzyme through hydrophobic interactions to form more stable complexes. Therefore, the realization of hypoglycemic function may be related to the number of phenolic hydroxyl groups in EGC and the number and ratio of non-polar amino acids in α-glucosidase and α-amylase. These interactions do not simultaneously change during EGC’s metabolism, nor do they affect its inhibition of α-glucosidase and α-amylase. Thus, this research can provide a theoretical basis for EGC as a novel food additive or glycoside hydrolase inhibitor to develop functional foods or hypoglycemic drugs. However, in this research, EGC’s hypoglycemic activity was only explored through *in vitro* experiments, making it difficult to reflect EGC’s mechanism on enzymes *in vivo*. Animal experiments should be performed to gain insight into the complex role of EGC *in vivo* in the future. Additionally, individual cases of acute hepato-toxicity from a large amount of green tea consumption have been reported in the literature, but the diverse composition and coingestants make it difficult to establish a clear causal relationship between green tea extract and reported cases of hepato-toxicity (48–50). Most published reports use EGCG concentrations ranging from 10 to 100 mmol/L (49, 51), but the range of concentrations used for EGC has not been clearly reported. As the homologous substance of EGCG, there is only about 35% of EGCG, so the theoretical safe dose of EGC is 3.5–35 mmol/L. However, this will not significantly impact the beneficial effects of tea and EGC on hypoglycemic.

## Data availability statement

The original contributions presented in this study are included in the article/supplementary material, further inquiries can be directed to the corresponding author.

## Author contributions

XW, ZM, JX, and HY planned for the research. ZM, YF, and XW performed the experiments. All authors have contributed in the research work and the write-up of this article.

## Footnotes

1
https://www.rcsb.org/

2
https://pubchem.ncbi.nlm.nih.gov/
